# Frequency of therapeutic drug monitoring in inpatient precision depression care and duration of hospitalization

**DOI:** 10.1038/s41398-026-04316-8

**Published:** 2026-08-01

**Authors:** Chantal Hampf, Jürgen Deckert, Sebastian Walther, Heike Weber, Stefan Unterecker, Maike Scherf-Clavel

**Affiliations:** 1 Department of Psychiatry, Psychosomatics and Psychotherapy, Center of Mental Health, University Hospital of Würzburg, Würzburg, Germany; 2 Institute of Clinical Epidemiology and Biometry, Julius-Maximilians-Universität Würzburg, Würzburg, Germany; 3 Department of Psychiatry and Psychotherapy, Social Foundation Bamberg, Teaching Hospital of the University of Erlangen, Bamberg, Germany

**Keywords:** Depression, Prognostic markers

## Abstract

In psychiatry, therapeutic drug monitoring (TDM) is routinely used to improve treatment safety and efficacy, yet its association with hospitalization duration and cost-effectiveness remains unclear. We hypothesized that the frequency of routinely requested TDM is associated with hospitalization duration. In this retrospective study, patients with major depressive disorder receiving TDM of antidepressants between 2015 and 2021 (*N* = 383) were analyzed. Inclusion criteria were a hospital stay ≥7 days and a first TDM request at admission. Regression analyses assessed associations between TDM frequency and hospitalization duration. Receiver operating characteristic analysis identified a minimum effective TDM frequency of 0.66/week, which was associated with a mean reduction in hospitalization duration of 21 days (*p* < 2.2*10^−^¹⁶). The upper effective threshold was approximated at 1.3/week. Higher TDM frequency was associated with more therapy adjustments within 7 days after TDM, including dose adaptations (*p* = 3.07*10^−^¹⁶) and drug initiation/discontinuation (*p* = 6.70*10^−6^). Drug-specific analyses for amitriptyline, venlafaxine, and mirtazapine confirmed these associations. Cost-effectiveness analysis demonstrated a 37% cost reduction for patients with TDM frequencies ≥0.66/week. In conclusion, frequent, clinically integrated TDM of antidepressants and timely treatment adaptations were associated with shorter hospitalization and reduced hospitalization costs. These findings suggest that at least biweekly TDM combined with subsequent treatment adaptations may represent a previously underappreciated strategy to improve clinical and economic outcomes in a tertiary referral center for severe major depression. Whether these findings are transferable to other inpatient and outpatient settings of depression care requires further studies.

## Introduction

Therapeutic drug monitoring (TDM) is established to provide precision medicine in psychiatry. Dosages can be individually adapted to provide the most effective treatment. The TDM task force of the working group on neuropsychopharmacology (Arbeitsgemeinschaft fuer Neuropsychopharmakologie und Pharmakopsychiatrie, AGNP) issued the first best- practice guidelines for TDM in psychiatry in 2004 [1]. In 2011, 2018, and 2026, the guidelines were updated and considerably extended to include a broad range of additional drugs [2–4]. Drug-specific therapeutic reference ranges (TRR) include lower limits of concentrations, below which a therapeutic response is unlikely to occur. An upper limit is included, above which further therapeutical improvement is improbable or which is linked to excessive adverse effects.

TDM is utilized with a high level of recommendation (level 1) for different antidepressant drugs, e.g. for amitriptyline. It is also recommended for others with a level 2 recommendation, including venlafaxine and mirtazapine. According to the AGNP TDM task force recommendations, TDM is mainly requested when clinical improvement is lacking, adverse effects occur despite recommended dosages, or adherence is questionable [2]. Similarly, the German national healthcare guidelines of depression, recommend TDM for non-responding patients [5]. Thus, TDM is neither applied routinely to all patients under psychopharmacological therapy, nor performed in a regular frequency.

Data on the cost-effectiveness of TDM are rare. Studies on the duration of inpatient treatment are available for citalopram, comparing low and high concentrations of citalopram [6]. Patients with high serum concentrations of citalopram (>50 ng/ml; mean duration 49 days) were on average 23 days shorter hospitalized than patients with low concentrations (<50 ng/ml; mean duration 72 days) [6]. Likewise, patients with TDM-guided antidepressant treatment with nortriptyline were discharged 6.1 days earlier and returned to work sooner (55.4 days) compared to treatment without TDM [7]. The introduction of TDM during selective serotonin reuptake inhibitor (SSRI) treatment reduced treatment costs by 16% [8]. Higher serum concentrations of citalopram and venlafaxine and their active metabolites have been reported to be associated with improved clinical outcomes and are predictive of later response in depression [9–11]. Finally, TDM can contribute to the prevention of adverse drug reactions by identifying excessively high serum concentrations associated with concentration-dependent toxicity. It thus enables timely dose adjustment before clinically apparent or serious complications occur, such as cardiac conduction abnormalities or arrhythmias. As a consequence, it can reduce healthcare costs by avoiding the need to manage toxicity-related complications [12, 13].

To address the cost-effectiveness of TDM, we previously reported that routinely requesting TDM for antidepressants upon hospital admission reduced inpatient treatment duration by a mean of 5 days [14]. To follow-up the results, we aimed to investigate whether, beyond the timing of TDM initiation, the frequency of TDM can also affect the duration of hospitalization. We hypothesize that frequent TDM requests support changes in pharmacotherapy as a mechanism to decrease the duration of hospitalization.

## Materials and methods

At the Department of Psychiatry, Psychosomatics and Psychotherapy of the University Hospital of Würzburg, a routine TDM service has been available since 2003, and serum samples from patients treated with psychotropic drugs are analyzed on request. According to the AGNP-TDM expert group consensus guideline, blood is drawn at trough concentration at steady-state [2]. In our study blood sampling was routinely performed in the morning prior to the next dose. Until 2019, serum concentrations of the drugs and their metabolites were determined using an online solid-phase extraction sample clean-up followed by an isocratic reversed-phase high-performance liquid chromatography (HPLC) method with either UV absorbance detection, or fluorescence detection. More recent samples (since 07/2019) were determined using liquid chromatography tandem mass spectrometry (LC-MS/MS) methods using MassTox^®^ TDM Serie A, provided by Chromsystems (Chromsystems Instruments & Chemicals GmbH, Gräfelfing, Germany) [15]. Daily calibrations and internal quality control samples ensure correct analytical results. The laboratory was certified by a quality control program [16]. The turnaround time for TDM results was typically 1–3 days in our institution. Since 2015, all TDM results have been systematically recorded in an electronic database.

We retrospectively assessed patients between 2015–2021 with TDM determinations of all antidepressant drugs for which therapeutic drug monitoring was available at our institution. The antidepressants included amitriptyline, escitalopram, mirtazapine, sertraline, nortriptyline, citalopram, clomipramine, doxepin, duloxetine, fluoxetine, fluvoxamine, imipramine, maprotiline, milnacipran, paroxetine, trazodone, trimipramine and bupropion. Analyses included patients with a major depressive disorder according to ICD-10. The minimum duration of hospital stay was set at 7 days for these analyses to exclude patients with a planned short stay in hospital, for example to perform a maintenance short-term electroconvulsive therapy (ECT) treatment. Similarly, patients who were discharged prematurely at own risk were excluded from the analyses. To overcome a bias due to timing of the first TDM, only patients with their first TDM at admission to hospital were included in the analysis (*N* = 383; Supplemental Fig. 1). Admission was defined as date of admission plus 2 days [14]. Consequently, all patients in the present analysis received TDM. The study therefore does not compare patients with versus without TDM, but rather examines the association between the frequency of TDM and duration of hospitalization within this cohort. Decisions on patients’ discharge were based on clinical judgment.

The retrospective analysis of clinical routine data was performed in accordance with a statement by the Wuerzburg ethics committee (20230720 03) and the principles of the Declaration of Helsinki.

### Statistical analyses

Statistical analyses were conducted in R v4.0.4 [17].

The primary outcome of this study was duration of hospitalization. Secondary outcomes included therapy adjustments following TDM and cost-related outcomes, in order to further explore potential mechanisms underlying and consequences of the observed associations.

For the analyses, the number of TDM requests per patient per week was calculated irrespective of the specific drug, as patients were not necessarily treated with the same medication throughout hospitalization. If two or more TDM requests for different drugs occurred on the same day, they were counted as a single request. Outliers (deviation ≥3 standard deviations (SD) from the mean) in duration of hospitalization were excluded from analyses.

After a first visual inspection of the association between the duration of hospitalization and frequency of TDM, a logarithmic, as well as an exponential regression analysis was applied to assess the significance of the association. Age at admission, sex of the patients and lithium comedication were included as covariates. The models were compared to receive the most accurate one, describing the data at best (Akaike information criterion (AIC), p-values, and the variance explained by the model). Multicollinearity was evaluated using variance inflation factors (VIFs) with a commonly accepted threshold of 5.

To evaluate the most effective frequency of TDM requests, duration of hospitalization was split into duration longer and shorter than the median of hospitalization. Given the non-parametric approach, the median was considered a more appropriate measure of central tendency. To investigate the association between TDM frequency and duration of hospitalization, Kruskal–Wallis tests were used. Receiver operating characteristic (ROC) analysis was used to define a threshold in TDM frequency that was associated with a time shorter than the median of hospitalization in the sample, using the ROCit, and pROC package in R [18, 19]. The sample then was split in patients with a frequency of TDM requests equal or higher than the threshold, and lower than the threshold. Duration of hospitalization was compared in the groups using Kruskal-Wallis test.

To approximate an upper threshold of TDM frequency beyond which no further reduction in hospitalization duration could be expected, we visually inspected the data and performed linear regression analyses restricted to patients with a TDM frequency ≥1. Subsequently, the analyses were repeated iteratively using progressively higher TDM frequency cutoffs (increments of 0.1) until the association between TDM frequency and duration of hospitalization was no longer statistically significant.

To investigate whether therapy adjustments due to TDM results are the driving factor for a decrease in duration of hospitalization, the association between the number of TDM analyses per week of hospitalization and the number of therapy adjustments (dose modification, drug withdrawal/addition) per week of hospitalization was investigated applying a linear regression analysis. Therapy adjustments were limited to adjustments that were done within 7 days after TDM to mainly include adjustments due to TDM results. Adjustments were defined as dose modifications of the respective drug, or discontinuation or start with an (additional) antidepressant, antipsychotic drug, or mood stabilizer. Age at admission and sex of the patients were included as confounders. Multicollinearity was evaluated using VIFs.

For drug-specific analyses, only patients with TDM measurements for the respective drug both at admission and at discharge were included. This approach was used to ensure that patients were continuously treated with the respective drug throughout hospitalization. For amitriptyline, venlafaxine, and mirtazapine a sufficiently large patient sample was available. Again, outliers (deviation ≥3 SD from the mean) in duration of hospitalization, as well as TDM frequency were excluded from analyses. After first visual inspection of the association between the duration of hospitalization and frequency of TDM, linear regression analysis was applied to assess the significance of the association. Age at admission, sex of the patients, and lithium comedication were included as confounders. To investigate if therapy adjustments due to TDM results are the driving factor for a decrease in duration of hospitalization, the association between the number of TDM analyses per week of hospitalization and the number of therapy adjustments (dose modification, drug withdrawal/addition) per week of hospitalization was investigated applying a linear regression analysis. Age at admission, and sex of the patients were included as confounders. Multicollinearity was evaluated using VIFs.

A cost approximation was performed to estimate potential differences in hospitalization-related expenses according to TDM frequency. Daily inpatient costs for psychiatric hospitalization were assumed to be €350. Mean hospitalization costs per patient were calculated by multiplying daily costs by the mean duration of hospitalization in patients with TDM frequency above and below the threshold frequency (0.66 TDM per week). Costs for TDM analyses were estimated based on a reimbursement of €52 per analysis using liquid chromatography–tandem mass spectrometry [20]. For patients with an effective TDM frequency, TDM-related costs were calculated assuming the upper effective frequency threshold of 1.3 TDM requests per week and a mean hospitalization duration of 34.1 days. This resulted in 6.37 TDM requests per patient. Total TDM-related costs were obtained by multiplying the number of TDM requests by the unit cost per analysis. Total mean costs per patient were calculated as the sum of hospitalization-related costs and TDM-related costs and compared between patients with lower and higher TDM frequency.

P-values were adjusted for multiple comparisons using the false discovery rate (FDR) according to the Benjamini–Hochberg procedure for the total number of analyses in the overall sample irrespective of the drugs (*n* = 7), as well as the drug-specific analyses (each *n* = 2), with an adjusted significance threshold of *p* ≤ 0.05. The analyses to approximate an upper threshold for TDM frequency was excluded from FDR correction, as this was an explorative approach.

## Results

### Patient sample

After applying all exclusion criteria, 383 patients were eligible for analysis. The mean age of the patients was 51.9 years (standard deviation, 17.0 years; range (min, max), 18.3 years, 91.9 years); 162 patients were male (42.3%) and 221 female (57.7%). The mean duration of hospitalization was 45.1 days (standard deviation (SD) 25.0 days; min 8 days; max 142 days), with a median duration of 41 days (interquartile range (25%–75%) 26.5–60 days). Prevalent diagnoses were as followed: Severe depressive episode without psychotic symptoms (F32.2; *N* = 41, 10.7%), severe depressive episode with psychotic symptoms (F32.3; *N* = 6, 1.57%), recurrent depressive disorder, current episode severe without psychotic symptoms (F33.2; *N* = 321, 83.8%), and recurrent depressive disorder, current episode severe with psychotic symptoms (F33.3; *N* = 15, 3.92%). 91 patients were co-medicated with lithium.

Within 7 days after TDM, the drug dose was modified at mean 2.01 (SD 1.62; min 0; max 10) times per patient, and a psychotropic drug (antidepressant, antipsychotic or mood stabilizer) was withdrawn or added at mean 0.9 (SD 1.06; min 0; max 7) times per patient. Relating to the number of TDM measurements (adjustment/number of TDM), mean number of dose adjustments within 7 days after TDM was 0.48 (SD 0.27; min 0; max 1), mean number of withdrawal or addition of a psychotropic drug was 0.23 (SD 0.24; min 0; max 1).

Mirtazapine was the most frequently monitored drug (482 determinations in 168 patients), followed by amitriptyline (412 determinations in 143 patients), and venlafaxine (295 determinations in 99 patients). Drugs, number of patients, number of TDM determinations, frequency of TDM requests (requests per week) and duration of stay in hospital are summarized in Table 1.

**Table 1:** Number of patients, TDM requests, dose of the drugs, concentration of the active moieties, frequency of TDM requests (number of requests per week), and duration of hospital stay for individual antidepressant drugs.

			Dose (mg)				Concentration [ng/ml]				N (TDM per patient)				Frequency of TDM requests [TDM request/week]				Duration of hospital stay [days]			
Drug	N(Patient)	N(TDM)	Mean	SD	Min	Max	Mean	SD	Min	Max	Mean	SD	Min	Max	Mean	SD	Min	Max	Mean	SD	Min	Max
**Amitriptyline**	143	412	105.6	57.4	16	750^a^	155.7	89.4	0	644	2.88	1.88	1	11	0.42	0.27	0.05	2.10	53.60	26.38	10	142
**Bupropion**	25	56	283.9	79.3	150	450	869.1	497.2	52	1948	2.24	1.54	1	7	0.38	0.19	0.11	1.00	45.52	23.49	14	108
**Citalopram**	18	19	27.2	13.7	10	60	50.5	31.2	0	106	1.06	0.24	1	2	0.17	0.17	0.05	0.78	64.56	35.08	9	142
**Clomipramine**	43	114	133.1	65.1	25	375	302.5	179.2	0	1063	2.65	1.62	1	7	0.44	0.34	0.08	1.75	53.67	26.36	8	119
**Doxepine**	32	60	107.4	45.2	0	200	93.3	79.6	0	379	1.88	1.01	1	5	0.46	0.48	0.08	2.63	46.63	33.91	8	142
**Duloxetine**	33	92	92.8	29.7	30	150	59.8	52.5	3	279	2.79	1.82	1	8	0.51	0.29	0.05	1.25	45.06	25.59	8	133
**Escitalopram**	58	117	15.2	6.7	2.5	40	37.7	28.5	0	231	2.02	1.15	1	5	0.37	0.28	0.05	1.40	49.53	27.97	11	142
**Fluoxetine**	8	9	29.4	11.5	15	40	205.6	136.5	16	482	1.13	0.35	1	2	0.18	0.07	0.07	0.25	52.63	26.23	28	98
**Fluvoxamine**	1	1	200.0		200	200	132.0		132	132	1.00				0.26				27.00			
**Maprotiline**	4	6	112.5	100.9	25	300	54.8	31.3	6	97	1.50	0.58	1	2	0.14	0.07	0.10	0.24	80.25	35.97	58	134
**Milnacipran**	14	29	88.8	39.9	25	150	70.4	55.2	0	247	2.07	0.92	1	4	0.46	0.37	0.06	1.24	44.00	24.62	15	108
**Mirtazapine**	168	482	32.3	14.6	7.5	75	44.4	28.2	0	228	2.87	1.79	1	11	0.56	0.35	0.07	1.75	45.96	26.91	8	142
**Nortriptyline**	3	6	75.0	22.4	50	100	106.7	23.4	86	132	2.00	1.00	1	3	0.30	0.23	0.13	0.57	59.67	41.88	34	108
**Paroxetine**	5	9	21.1	6.0	10	30	53.4	67.6	8	229	1.80	1.30	1	4	0.39	0.13	0.25	0.57	30.40	12.46	16	49
**Sertraline**	51	108	127.3	64.1	25	300	56.2	56.3	0	327	2.12	1.35	1	7	0.37	0.29	0.07	1.56	50.73	24.50	9	105
**Trazodon**	23	62	130.7	58.2	5	300	769.1	451.7	48	2605	2.70	1.55	1	7	0.42	0.33	0.08	1.32	54.22	17.04	25	90
**Trimipramine**	6	14	158.9	162.5	25	700	244.1	189.5	85	840	2.33	1.75	1	5	0.40	0.33	0.09	0.95	49.17	19.17	28	80
**Venlafaxine**	99	295	234.6	110.2	37.5	1100^a^	355.1	213.0	0	1288	2.98	1.99	1	9	0.50	0.36	0.08	2.19	47.97	22.73	9	119

*N* number, *TDM* therapeutic drug monitoring, *SD* standard deviation, *Min* minimum, *Max* maximum;

^a^Patients with 750 mg amitriptyline and 1100 mg venlafaxine respectively had taken this dose in the context of a suicide attempt. Next highest doses were for amitriptyline 300 mg and for venlafaxine 450 mg.

### Association between frequency of TDM and duration of hospitalization

TDM requests after admission had a mean of 3.9 (SD, 2.1; range, 1–13; median, 4; IQR, 2–5) during hospitalization. The average frequency (TDM requests per week) was 0.69 (mean; SD, 0.30; range, 0.11–1.87; median, 0.65; IQR, 0.5–0.81). The distribution of TDM request frequencies for the overall sample are shown in Supplemental Fig. 2.

After visual inspection, patients with a TDM request frequency deviating more than 3 standard deviations from the mean, were excluded from analyses (*N* = 5).

Exponential regression analysis showed that duration of hospitalization decreased with increasing frequency of TDM requests (*p* < 2.2*10^−16^; p(FDR) < 3.08*10^−16^; ß = −1.14; 95% CI = −1.32–−0.96; AIC = 576.23). No evidence of multicollinearity was detected.

Similarly, logarithmic regression analysis showed that duration of hospitalization decreased with increasing frequency of TDM requests (*p* < 2.37*10^−16^; *p*(FDR) < 3.08*10^−16^; ß = −22.43; 95% CI = −27.56–−17.29; AIC = 3465.46). No multicollinearity was detected. In neither of the models age, sex or lithium intake were associated with duration of hospitalization.

Comparison of the AIC values, corresponding p-values, and the proportion of variance in hospitalization duration explained by each model (exponential model: 29.8% (adjusted R^2^), logarithmic model: 17.8%) indicated that the exponential model provided the best fit to the data (Supplemental Fig. 3).

### Threshold in TDM frequency for a shorter time of hospitalization

TDM frequency was associated with duration of hospitalization longer and shorter than the median duration (42 days; Kruskal-Wallis test, *p* < 2.2*10^−16^, *p*(FDR) < 3.08*10^−16^, Supplemental Fig. 4).

ROC analysis (*p* < 2.2*10^−16^, *p*(FDR) < 3.08*10^−16^) revealed that a duration of hospitalization <42 days could be expected for a TDM request frequency of ≥0.66/week. For this value, predictive validity exhibited 74.7% specificity and 67.4% sensitivity. The area under the ROC curve was 0.750 (95% CI 0.70–0.80) (Supplemental Fig. 5).

Follow-up analyses demonstrated 21.3 days shorter hospitalizations in patients with a TDM frequency of ≥0.66/week, compared to those with lower TDM frequency (duration of hospitalization; <0.66 TDM/week: mean, 55.4; SD, 24.4; ≥0.66 TDM/week: mean 34.1; SD, 20.6; Kruskal-Wallis test, *p* < 2.2*10^−16^; p(FDR) < 3.08*10^−16^) (Supplemental Fig. 6).

**Fig. 1:**
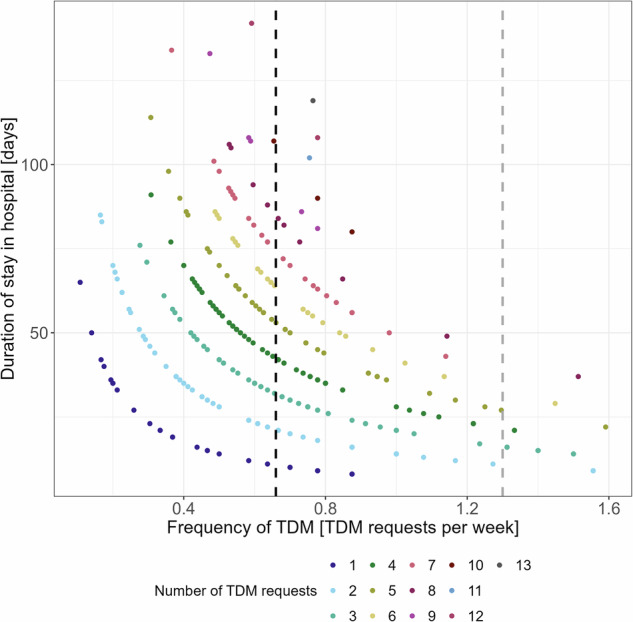
Association between duration of hospitalization and TDM frequency. Association is shown including the threshold (ROC analysis) in TDM frequency that was associated with a decrease in hospitalization (black dashed line), as well as the approximated upper value (grey dashed line) above that no decrease in duration of hospitalization may be expected.

To approximate a potential upper threshold of TDM request frequency beyond which no additional reduction in hospitalization duration was observed, the data were initially assessed by visual inspection (Fig. 1). Subsequently, a subset of patients with a TDM request frequency >1/week (*N* = 42) was analyzed to assess whether hospitalization duration continued to decrease at higher TDM frequencies. As a significant association was observed, linear regression analyses were iteratively repeated using progressively higher TDM frequency thresholds (increments of 0.1) until no significant association between TDM request frequency and hospitalization duration remained. For TDM frequencies >1.3 requests/week (*N* = 20, *p* = 0.295), no significant association was detected, suggesting that no further reduction in hospitalization duration may be expected beyond this threshold (Fig. 1).

### TDM analyses and therapy adjustments

**Fig. 2:**
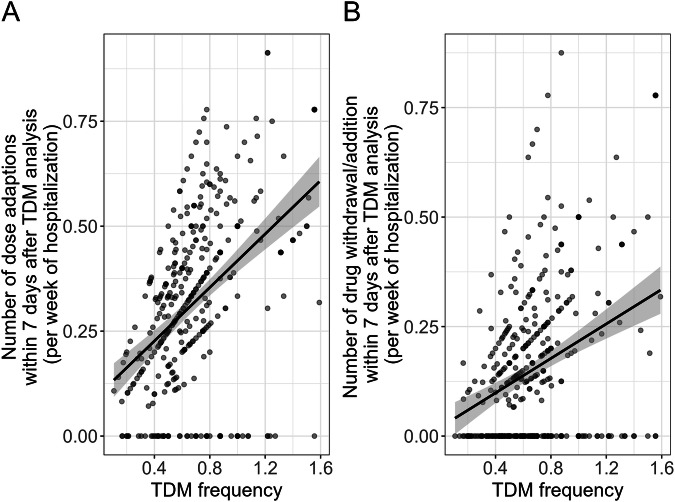
Association between TDM frequency and therapy adjustments. Number of TDM requests per week of hospitalization (TDM frequency) was positively associated with **A** number of dose adjustments (*p* = 3.07*10^−16^), and **B** number of drug withdrawal/addition (*p* = 6.70*10^−6^) within 7 days after TDM analysis per week of hospitalization. Point transparency reflects local data density, with darker points indicating a higher number of overlapping observations.

Number of TDM analyses per week of hospitalization (TDM frequency) was associated with number of dose adjustments per week of hospitalization (*p* = 3.07*10^−16^; p(FDR) = 3.58*10^−16^; ß = 0.57; CI = 0.44–0.70), as well as with number of drug withdrawal/addition per week of hospitalization (*p* = 6.70*10^−6^; p(FDR) = 6.70*10^−6^; ß = 0.35; CI = 0.20–0.50) within 7 days after TDM. With increasing number of TDM frequency, the number of therapy adjustments increased (Fig. 2). No evidence of multicollinearity was detected. Age or sex were not associated with TDM frequency.

### Drug specific analyses

#### Amitriptyline

In total, 71 patients with TDM of amitriptyline at admission were available, of whom 22 also had TDM measurements at discharge. *N* = 6 patients were co-medicated with lithium. Demographic data are reported in Tables 2 and 3.

**Table 2:** Demographic data of the patients included in the drug-specific analyses.

	Amitriptyline	Venlafaxine	Mirtazapine
**N=**	22	34	60
**Age (mean** **±** **sd) [years]**	56.7 ± 10.2	47.8 ± 14.0	59.2 ± 16.6
**Male/Female (N)**	10/12	19/15	28/32
**Duration of inpatient stay [days]**			
**mean** **±** **sd; min; max**	48.8 ± 28.6; 16; 107	45.9 ± 21.7; 16; 88	35.6 ± 19.0; 8; 81
**median; 25–75% IQR**	39; 29.25–70.00	43.0; 29.2–63.0	35; 21.0–48.0
**Prevalent diagnoses**			
**F32.2**	4.5%	5.9%	16.7%
**F32.3**	4.5%		1.7%
**F33.2**	81.8%	94.1%	76.7%
**F33.3**	9.1%		4.9%
**Number of TDM requests**			
**mean** **±** **sd; min; max**	4.3 ± 2.2; 2; 10	4.3 ± 1.8; 2; 8	3.7 ± 1.6; 1; 9
**median; 25**–**75% IQR**	4.0; 2–5	4; 3–5	4; 2.8–5.0
**Frequency of TDM requests [request per week]**			
**mean** **±** **sd; min; max**	0.68 ± 0.24; 0.38; 1.22	0.72 ± 0.27; 0.28; 1.33	0.84 ± 0.34; 0.21; 1.75
**median; 25**–**75% IQR**	0.6; 0.53–0.85	0.65; 0.54–0.88	0.78; 0.6–1.0

*sd* standard deviation, *min* minimum, *max* maximum, *IQR* interquartile range.

**Table 3:** Cost-effectiveness analysis for patients with an effective TDM frequency (0.66–1.3) compared to patients with a TDM frequency below 0.66.

	Patients with a TDM frequency < 0.66	Patients with a TDM frequency of ≥ 0.66
**Mean duration of hospitalization [days]**	55.4	34.1
**Daily costs for psychiatric hospitalization [€]**	350	350
**Approximated costs per patient for psychiatric hospitalization [€]**^a^	19 390	11 935
**Estimated costs per TDM analysis [€]**^b^	52	52
**Estimated number of TDM requests**^c^	0	6.37 (34.1/7days = 4.9 weeks; 4.9 x 1.3 = 6.37)
**Approximated costs per patient for TDM analyses [€]**^d^	0	331.24
**Overall costs per patient [€]**^e^	19 390	12 266.24
**Potential cost benefit per patient [€]**	7 123.76 (36.76%)	

^a^calculation: duration of hospitalization x daily costs for hospitalization;

^b^TDM analyses using liquid chromatography–mass spectrometry/mass spectrometry;

^c^for patients with a TDM frequency of 0.66 or higher the highest effective TDM frequency (1.3/week) was assumed for calculation to assume the highest possible costs, for patients with a TDM frequency below 0.66 no TDM was assumed for calculation to assume the lowest possible costs;

^d^calculation: number of TDM requests x 52€;

^e^calculation: approximated costs per patient for psychiatric hospitalization + approximated costs per patient for TDM analyses.

**Fig. 3:**
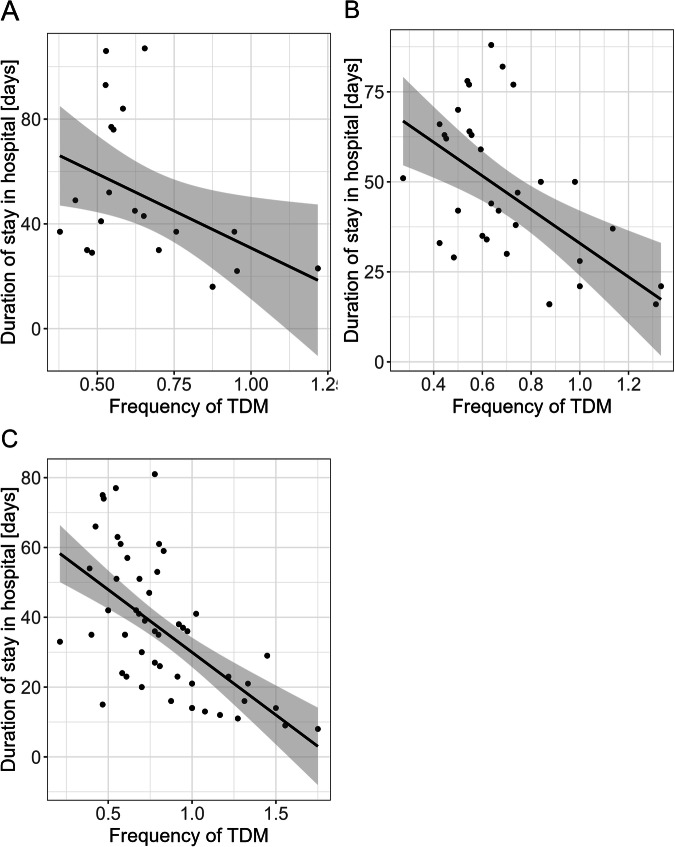
Duration of hospitalization was associated with frequency of TDM. Duration of hospitalization was shorter in patients for whom TDM was requested more frequently for amitriptyline **A** (linear regression, *p* = 0.03; also, in correlation analysis, frequency of TDM as associated with duration of hospitalization (Spearman correlation, *p* = 0.03)), venlafaxine **B** (linear regression, *p* = 1.9*10^−4^; also, in correlation analysis, frequency of TDM as associated with duration of hospitalization (Spearman correlation, *p* = 4.1*10^−4^)), and mirtazapine **C** (linear regression, *p* = 6.0*10^−8^; also, in correlation analysis, frequency of TDM as associated with duration of hospitalization (Spearman correlation, *p* = 8.4*10^−9^)). Black line, association plot; grey area, 95% confidence interval.

Duration of hospitalization decreased with increasing frequency of TDM requests (linear regression analysis; *p* = 0.03; p(FDR) = 0.045; ß = −56.53; 95% CI −111.37–−1.700)) (Fig. 3). Age, sex, or lithium intake were not associated with duration of hospitalization. No evidence of multicollinearity was detected.

TDM frequency was associated with number of dose adjustments (*p* = 5.83*10^−4^; p(FDR) = 1.75*10^−4^; ß = 0.76; CI = 0.38–1.13) within 7 days after TDM per week of hospitalization. With increasing number of TDM analyses, number of therapy adjustments increased (Supplemental Fig. 7). No evidence of multicollinearity was detected. Age, sex and number of addition/withdrawal of a psychotropic drug within 7 days after TDM per hospitalization week were not associated with TDM frequency.

#### Venlafaxine

In total, 93 patients with TDM of venlafaxine at admission were available, of whom 34 also had TDM measurements at discharge. *N* = 8 patients were co-medicated with lithium. Demographic data are reported in Table 2.

Duration of hospitalization decreased with increasing frequency of TDM requests (linear regression analysis; *p* = 2.7*10^−4^; p(FDR) = 8.1*10^−4^; ß = −49.27; 95% CI = −73.59–−24.95) (Fig. 3). No evidence of multicollinearity was detected. Age, sex, or lithium intake were not associated with duration of hospitalization.

TDM frequency was associated with number of dose adjustments (*p* = 6.90*10^−4^; p(FDR) = 0.001; ß = 0.91; CI = 0.42–1.40) within 7 days after TDM per hospitalization week. With increasing number of TDM frequency, number of dose adjustments increased (Supplemental Fig. 8). No evidence of multicollinearity was detected. Age, sex and number of addition/withdrawal of a psychotropic drug within 7 days after TDM per hospitalization week were not associated with TDM frequency.

#### Mirtazapine

In all, 154 patients with TDM of mirtazapine at admission were available, of whom 62 also had TDM measurements at discharge. A number of 2 patients were outliers in duration of hospitalization and, therefore, excluded for analyses. *N* = 9 patients were co-medicated with lithium. Demographic data of the included patients are reported in Table 2.

Duration of hospitalization decreased with increasing frequency of TDM requests (linear regression analysis; *p* = 3.57*10^−8^; p(FDR) = 1.07*10^−7^; ß = −38.48; 95% CI = −50.53–−26.43) (Fig. 3). No evidence of multicollinearity was detected. Age, or sex were not associated with duration of hospitalization.

TDM frequency was associated with addition/withdrawal of a psychotropic drug (*p* = 0.009; p(FDR) = 0.014; ß = 0.59; CI = 0.15–1.03) within 7 days after TDM per hospitalization week. With increasing number of TDM frequency, number of dose adjustments increased (Supplemental Fig. 9). No evidence of multicollinearity was detected. Age, sex and number of dose adjustments within 7 days after TDM per hospitalization week were not associated with TDM frequency.

### Cost-effectiveness analysis

Assuming daily costs of at least €350 for psychiatric hospitalization, mean costs per patient were €19 390 for those with a TDM frequency below 0.66, and €11 935 for patients with a TDM frequency of 0.66 or higher. Thus, a mean cost reduction of €7 123 (37%) per patient may be expected with a TDM frequency of 0.66 or higher. Considering estimated costs of €52 per TDM using liquid chromatography–mass spectrometry/mass spectrometry [20], even at the highest effective TDM frequency (1.3/week) costs for TDM (€331.24) will be lower than costs of one day in hospital. In sum, for patients with an effective TDM frequency, mean costs for duration of hospitalization potentially will be about €12 265 (€11 935 + €330), whereas for patients with lower TDM frequency, mean costs may be €19 320. Thus, data demonstrate that frequent TDM requests with accompanying clinical adjustment (dose adjustment, addition/withdrawal of a drug) will reduce overall mean costs (Table 2).

## Discussion

In this retrospective analysis of clinical routine data, we followed-up previously reported data [14] by investigating the association between duration of hospitalization and frequency of TDM requests (number of TDM requests per week) in patients suffering from major depressive disorder. Duration of hospitalization decreased with increasing TDM request frequency, both across all drugs and in drug-specific analyses for amitriptyline, venlafaxine, and mirtazapine. At least 0.66 TDM requests/week (irrespective of the drug) were associated with a reduction in hospitalization duration of approximately 21 days compared to patients with less frequent TDM requests. The number of TDM analyses per week of hospitalization was positively associated with both the number of dose adjustments and the number of psychotropic drug initiations/discontinuations within 7 days after TDM. This indicates that therapy adjustments prompted by TDM results contribute to the observed reduction in hospitalization duration.

Studies have reported enhanced response rates and fewer adverse effects when antidepressant therapy is individualized by TDM [11, 13, 21, 22]. Our findings extend this evidence by demonstrating a measurable impact on healthcare utilization. To the best of our knowledge, this is the first study reporting an association between frequency of TDM requests and duration of hospitalization: Duration of hospitalization decreased with increasing TDM frequency. To minimize potential bias related to the timing of TDM requests, only patients with TDM performed at admission were included in the analyses, as we had previously shown that TDM at admission was associated with shorter hospitalization duration [14]. We suggest that therapy adjustments due to TDM analyses (within 7 days after TDM) are the factor underlying the reduction in hospitalization duration observed in patients with an effective TDM frequency. Another mechanism may be that frequent TDM may enhance patient adherence, as patients are aware that serum levels are monitored regularly.

To find a threshold in the frequency of TDM requests in order to predict a decreased duration of hospitalization, we stratified patients into two groups regarding the duration of hospitalization. For the median split a threshold of 0.66 requests per week were found. Comparing duration of hospitalization in patients with TDM request frequency of equal and more vs. less often than 0.66/week, the threshold was associated with an about 21 days shorter hospitalization time. We approximated an upper threshold of 1.3 TDM requests per week, beyond which no further reduction in duration of hospitalization may be expected. Thus, shorter hospitalization duration was associated with a TDM request frequency of at least 0.66 requests per week, whereas no additional reduction was observed beyond 1.3 requests per week. In the absence of prior studies defining an optimal TDM frequency, our results constitute the first demonstration of the benefit of regular TDM. In drug-specific analyses for amitriptyline, venlafaxine and mirtazapine, we only included patients who received TDM for the respective drug both at admission and at discharge. This criterion was applied to ensure a continuous treatment with the respective drug. For amitriptyline, venlafaxine, as well as mirtazapine, TDM frequency was significantly associated with duration of hospitalization, consistent with the results considering all drugs.

In clinical routine treatment of major depression, response rates for treatment with amitriptyline, venlafaxine, and mirtazapine are 53% (8 weeks), 45% (8 weeks) and 43% (6 weeks), respectively [23–25]. For venlafaxine, remissions rates increased from 9% in week 2, to 15% in week 3, 27% in week 4, 38% in week 6, and 45% in week 8 [24]. Similarly, for mirtazapine, remission rates increased from 13% in week 2, to 33% in week 4, to 43% in week 6 [25]. However, data showed that in general only 40% to 45% of patients respond within 2 to 3 months of starting a first-line treatment [26]. Applying TDM at early time points can be expected to increase the remission rates earlier by earlier changes in treatment strategy. Adding augmentation strategies enhance response/remission rates in treatment resistant depression [27]. For example, adding lithium to treatment-refractory patients treated with amitriptyline produced a significant and clinically meaningful improvement from day 7 to 12 [28]. According to our data, we suggest that using frequent TDM may shorten the time to add augmentation strategies and thus shorten time to remission. Due to the limited sample size, further analyses, i.e. ROC analyses were not conducted for amitriptyline, venlafaxine and mirtazapine separately. In drug specific analyses the TDM frequency, however, was positively associated with the number of dose adjustments (amitriptyline, venlafaxine), and number of addition/withdrawal (mirtazapine) of a psychotropic drug within 7 days after TDM per hospitalization week. Thus, again, we suggest that therapy adjustments due to TDM analyses (within 7 days after TDM) are the factor underlying the decrease in duration of hospitalization.

The relatively frequent use of amitriptyline in our cohort likely reflects the specific clinical setting of a university hospital as a tertiary referral center, where patients with complex and difficult-to-treat depression are overrepresented. Many of these patients have already received first-line treatments without sufficient response. This necessitates the use of second- or third-line treatment strategies, including tricyclic antidepressants, to achieve a response allowing discharge. As a consequence, amitriptyline remains clinically relevant as the most effective antidepressant in recent metaanalyses [29].

Reducing duration of hospitalization is advantageous not only for the patient, but also for the health system, as this is associated with reduced costs. For patients with an effective TDM frequency, mean costs for duration of hospitalization were estimated to be about €12 265, whereas for patients with lower TDM frequency, mean costs were estimated be €19 390. Requesting TDM frequently with accompanying clinical adjustment (dose adjustment, addition/withdrawal of a drug) will reduce overall mean costs by €7 123.76 per patient or by 37%.

### Strengths and limitations

We studied the duration of hospitalization in retrospective analyses of clinical routine data. Treatment within standardized programs for affective disorders in Würzburg is elective and may therefore introduce a selection bias, as participants may not be representative of all patients with affective disorders. However, these programs apply well-established treatment approaches in a consistent and standardized manner, which enhances internal validity. The analysis included a relatively small number of analysed patients (*n* = 383). This limitation is partly attributable to the strict inclusion criteria applied, which, while reducing sample size, enhance the internal validity and robustness of the findings. Due to the applied inclusion criteria, the investigated cohort consisted of patients with comparatively severe depression. This should be considered when interpreting the results, as the generalizability to less severely affected patient populations may be limited. There is a potential for confounding by indication, as patients requiring more frequent TDM may represent a subgroup with more complex clinical conditions (e.g., non-adherence, treatment resistance, or adverse effects). Notably, despite this potential bias, higher TDM frequency was associated with shorter hospitalization duration, supporting the potential clinical utility of frequent TDM in this subgroup of severe major depression. A subgroup (*n* = 53) of the patients also took part in the GePard study [30]. Only in them standardized rating scales were applied by trained members of the staff over the 7-year sampling period with a risk for differences in interindividual ratings. However, decisions with regard to discharge, the primary outcome variable of the present analysis, were based on clinical judgment rather than standardized rating scales. The discharge from the hospital was used as a surrogate marker for clinical response, as this is done when at least partial remission was achieved. Therefore, the time to discharge may indicate the duration of the necessary treatment. Although, thus, there was no formal assessment of response and remission using expert rating scales for the analysis, the clinical routine procedures offer a naturalistic perspective with great potential for generalizability. To prevent a bias due to patients that discharged at own risk without adequate clinical response, these patients were excluded from analyses. In the analyses, only patients with TDM at admission were included. While this procedure limited the sample size, it increased the robustness of our results by minimizing bias related to TDM timing. Antidepressants may be prescribed at different doses across indications. However, all patients were diagnosed with a depressive episode and treated for this indication, which reduces this source of variability in our sample. In drug-specific analyses the sample size was even further limited due to the requirement of TDM measurements at both admission and discharge; however, these criteria also strengthened the robustness of the analyses. The upper limit of an effective TDM frequency was approximated by using an exploratory approach by repeating regression analyses until the association was not significant anymore. Consequently, this threshold must be interpreted with caution as it may be too high. To distinguish if the higher TDM frequency is the cause for shorter hospitalization, or if the higher frequency is a result of actions following TDM (e.g. dose adjustment, comedication, drug switch), we included information of changes in pharmacotherapy e.g. dose adjustment, or switch of a drug in the analyses. We did not control for any other additional therapies, for example antidepressant combination or augmentation strategies, psychotherapy, ECT or repetitive transcranial magnetic stimulation (rTMS). Also, we did not differentiate between different therapy adjustments such as switching and augmentation. In consequence, we cannot estimate the contribution of the effect of combination therapies. Clinical response is multifactorial, and thus, duration of hospitalization may be a result of combination of different antidepressant therapies. We also did not systematically account for antidepressant dosages or co-medications, both of which may influence pharmacokinetics and clinical outcomes and thus could represent potential confounding factors. Additionally, discontinuation or start with other drugs (beside psychotropic drugs) were not included in therapy adjustment definition. However, these drugs may also influence serum concentrations, therefore, given the rational for adjustment after TDM. In addition, we did not consider if serum concentrations were within the therapeutic reference range or not. To the best of our knowledge, this is the first analysis considering these numbers of patients and antidepressant drugs in context of duration of hospitalization. A further concern is the high frequency of TDM measurements in some patients (>1/week), possibly also including sampling prior to steady-state conditions. This reflects real-world clinical practice, where TDM reflects continuous therapy decisions and is used to guide ongoing treatment adjustments. However, our data clearly show that TDM frequencies >1.3 per week do not contribute to a shorter hospital stay in line with established TDM guidelines. The main analyses were not restricted to specific drugs. While this may have introduced heterogeneity due to potential treatment changes over time, it enabled us to address our primary research question regarding the impact of TDM frequency on hospitalization duration in a more generalizable manner. Additional analyses were performed for the most frequently prescribed antidepressants (amitriptyline, venlafaxine, and mirtazapine) to assess potential temporal changes in prescribing practices over the years that could influence the results. No significant changes over time were observed for these compounds, suggesting largely stable prescribing practices during the study period. TDM data of all antidepressant drugs were included, even if evidence for doing TDM may be poor for some drugs. However, results were confirmed in the mirtazapine, venlafaxine, and amitriptyline samples, for which robust evidence supports the clinical use of TDM. Measured concentrations may not consistently represent true trough levels, particularly for antidepressants administered once daily in the evening. However, recommended TDM concentrations have been defined using this sampling method. While the high level of expertise in TDM at our center may limit the generalizability of the results to settings with less established TDM practices, it may at the same time illustrate the potential benefits achievable with broader adoption and increasing expertise in TDM in routine practice.

### Conclusion

As no previous study has established an optimal frequency for TDM, our findings provide the first evidence of a benefit from regular TDM. These data are of particular importance for clinical practice. Regularly requesting TDM for antidepressant drugs with accompanying clinical adjustment (dose adjustment, addition/withdrawal of a drug) decreased the duration of hospitalization in inpatients with major depressive disorder. We approximated an effective TDM frequency of at least 0.66 times per week. This may reduce duration of hospitalization by a mean of 21 days, increasing the patients’ well-being while substantially decreasing healthcare costs. These results suggest that frequent, clinically integrated TDM may represent a previously underappreciated strategy to improve both clinical and economic outcomes in severe major depressive disorder. In summary, we recommend that patients with severe major depressive disorder in inpatient settings who receive antidepressant medication shall undergo regular TDM - ideally at least biweekly with subsequent treatment adjustments. Further studies are necessary to evaluate if these findings are transferable to other inpatient and outpatient settings of depression.

## Supplementary information

Electronical Supplement

## Data Availability

The data that support the findings of this study are available from the corresponding author upon reasonable request. The data are not publicly available because they contain information that could compromise the privacy of research participants.
