# Supplementary material for: Frequency of therapeutic drug monitoring in inpatient precision depression care and duration of hospitalization
Source: Transl Psychiatry. 2026 Aug 1;16:387. doi: 10.1038/s41398-026-04316-8 (PMC13427846; doi:10.1038/s41398-026-04316-8)

Electronical Supplementary Material

to

Original Research Article

Frequency of therapeutic drug monitoring in inpatient precision depression care and duration of hospitalization

Short running title: Drug monitoring in precision depression care

Chantal Hampf[1], Jürgen Deckert, MD[1,2], Sebastian Walther, MD[1], Heike Weber, PhD[1], Stefan Unterecker, MD[1,3], Maike Scherf-Clavel, PhD[1#]

[1] Department of Psychiatry, Psychosomatics and Psychotherapy, Center of Mental Health, University Hospital of Würzburg, 97080 Würzburg, Germany

[2] Institute of Clinical Epidemiology and Biometry, Julius-Maximilians-Universität Würzburg, Würzburg, Germany

[3] Department of Psychiatry, Psychosomatic Medicine and Psychotherapy, Social Foundation Bamberg, Teaching Hospital of the University of Erlangen, Germany

[#]Corresponding author:

PD Dr. rer. nat. Maike Scherf-Clavel
Department of Psychiatry, Psychosomatics and Psychotherapy
University Hospital of Würzburg
Margarete-Höppel-Platz 1
97080 Würzburg, Germany
Tel.: +49/931/201 77260
Fax: +49/931/201 77262
E-Mail: Scherf_M@ukw.de

Supplemental Figure 1

Flow chart illustrating participant selection, inclusion, and exclusion criteria.

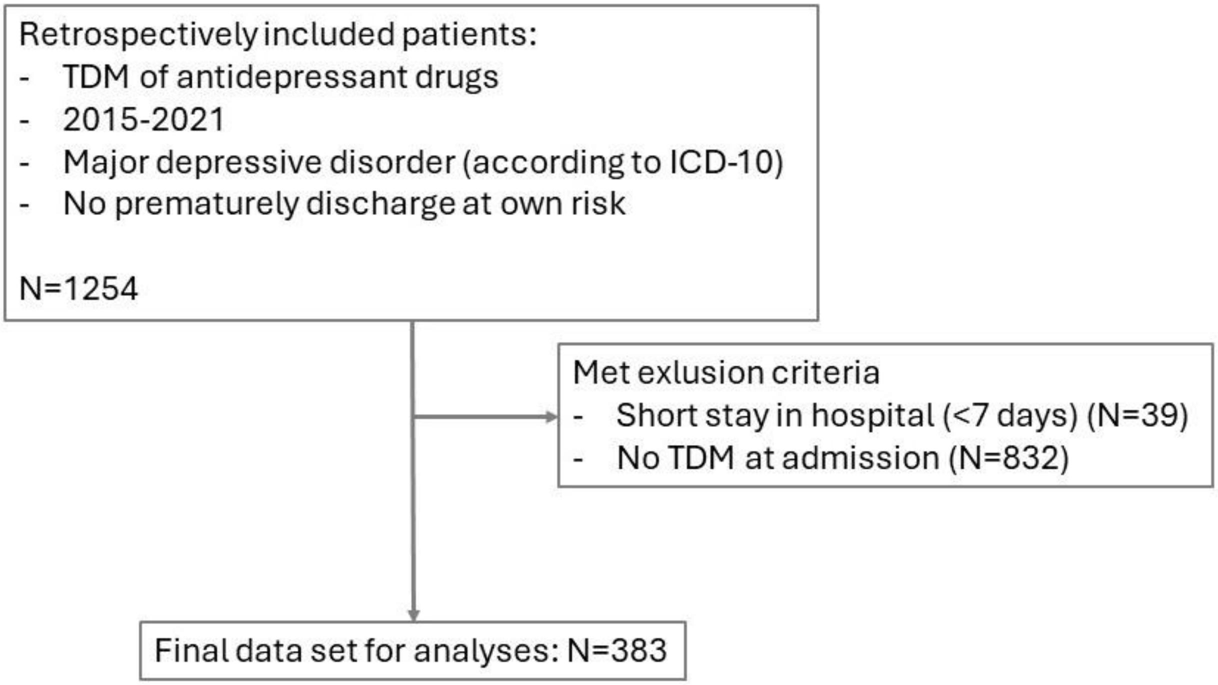

Supplemental Figure 2

Frequencies of TDM requests in the overall sample. After visual inspection, patients with TDM request frequency deviating more than 3 standard deviations from the mean, were excluded from analyses.

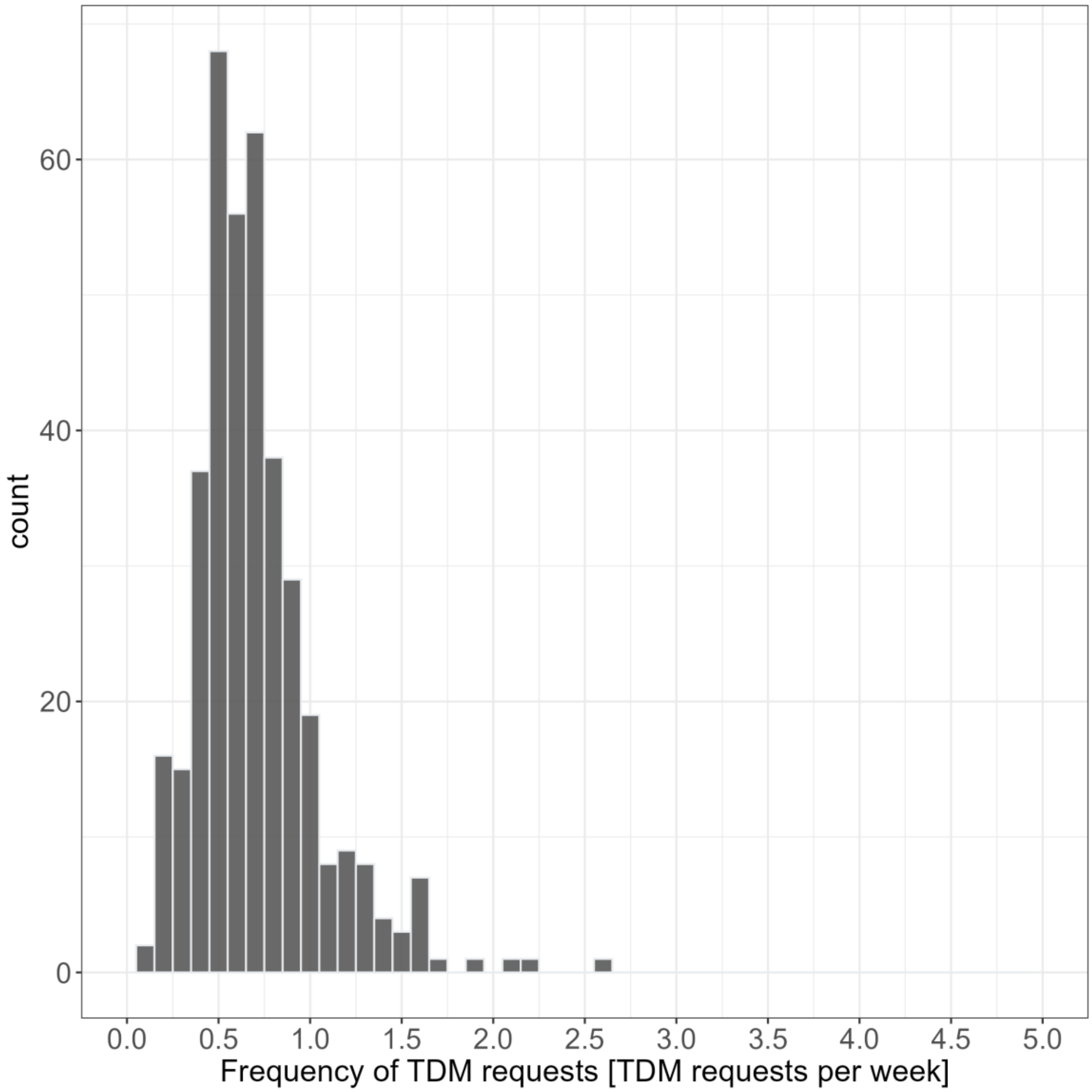

Supplemental Figure 3

Duration of hospitalization was associated with frequency of TDM. Duration of stay in hospital was shorter in patients for whom TDM was requested more frequently (exponential regression, $p<2.2*10^{-16}$). Black line, association plot (exponential association); grey area, 95% confidence intervals.

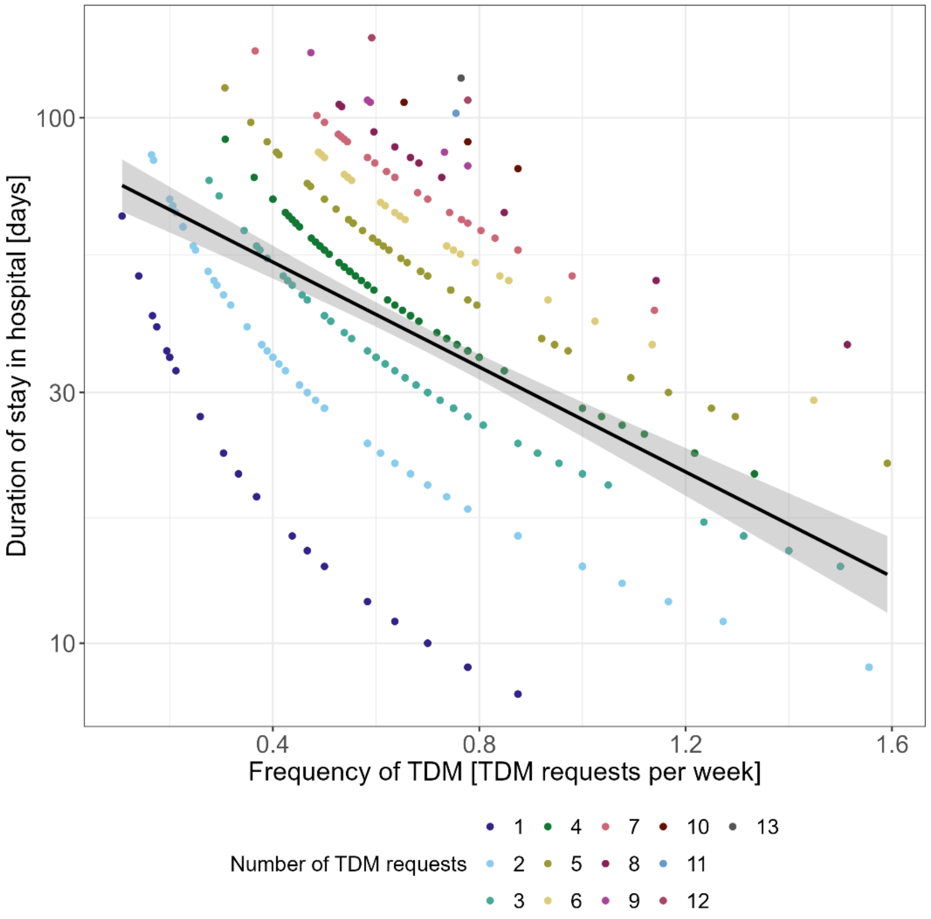

Supplemental Figure 4

Frequency of TDM requests was higher in patients with a shorter duration of hospitalization than the median duration (Kruskal-Wallis test, $p<2.2*10^{-16}$).

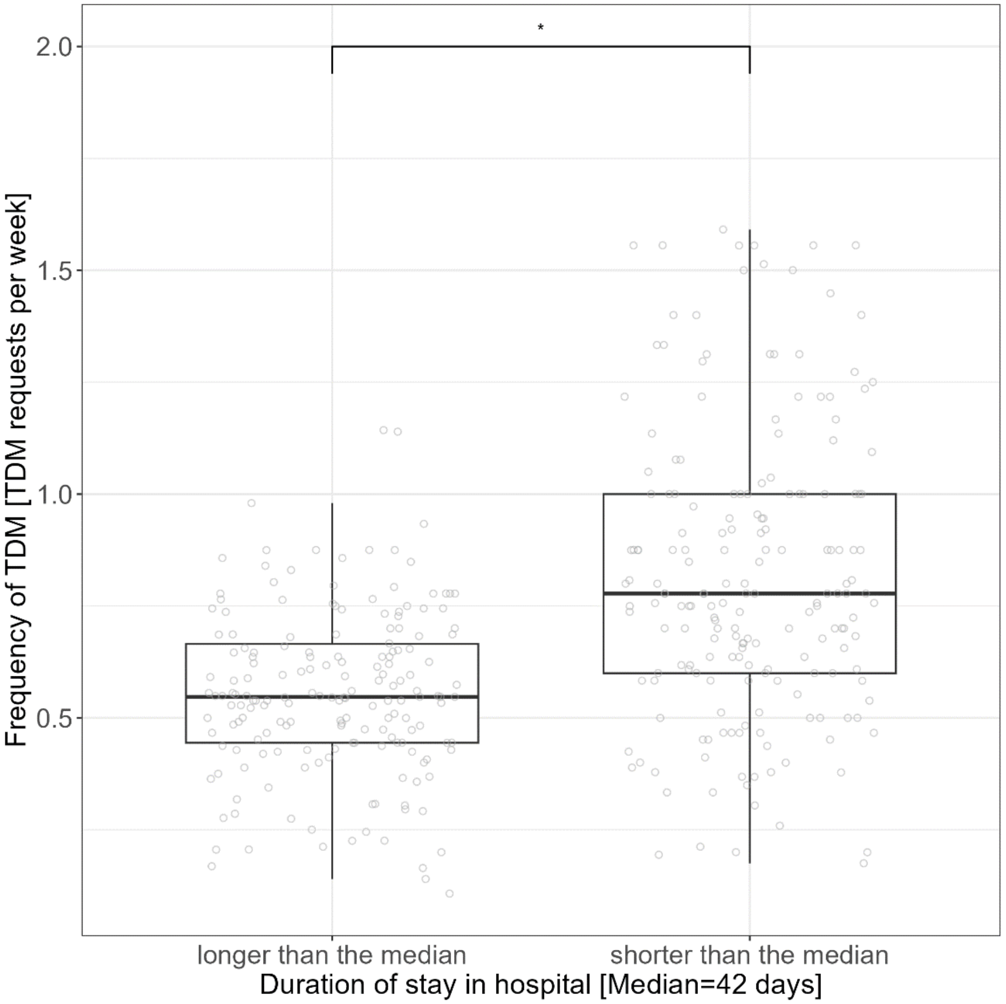

Supplemental Figure 5

ROC curve of the frequency of TDM requests associated with duration of hospitalization (longer/shorter than the median duration (42 days)). Frequency was computed as 0.66/week (74.7% specificity, 67.4% sensitivity). The area under the ROC curve was 0.75 (95% CI 0.70-0.80).

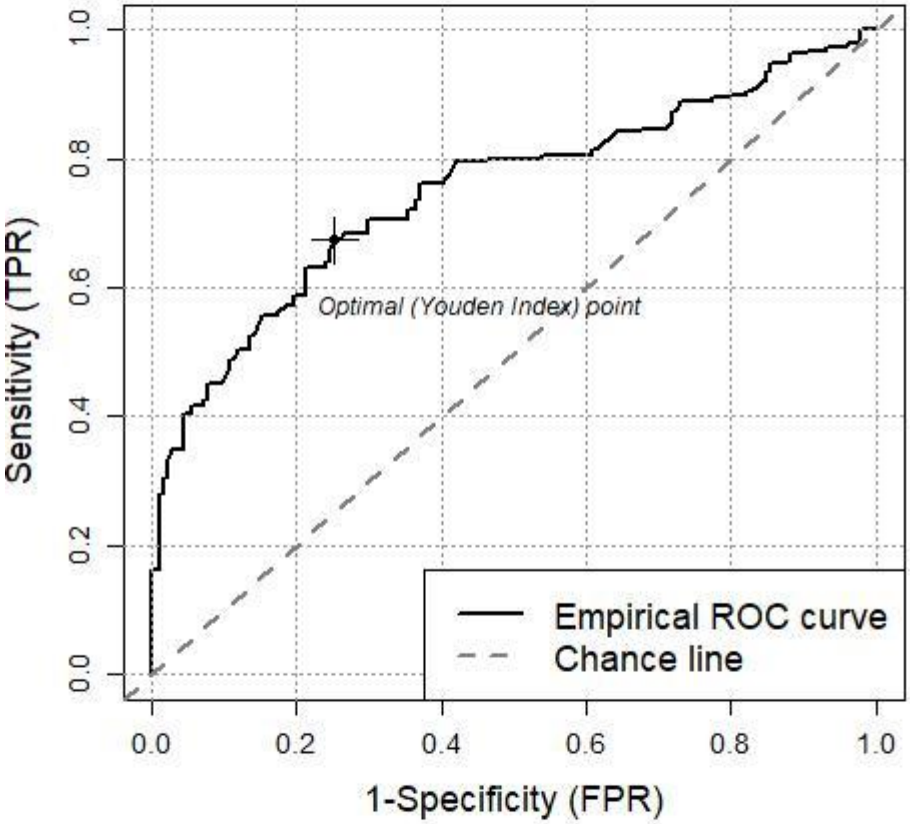

Supplemental Figure 6

Duration of stay in hospital was shorter in patients for whom TDM was requested 0.66 times or higher compared to patients for whom TDM was requested less often (Kruskal-Wallis test; $p<2.2*10^{-16}$).

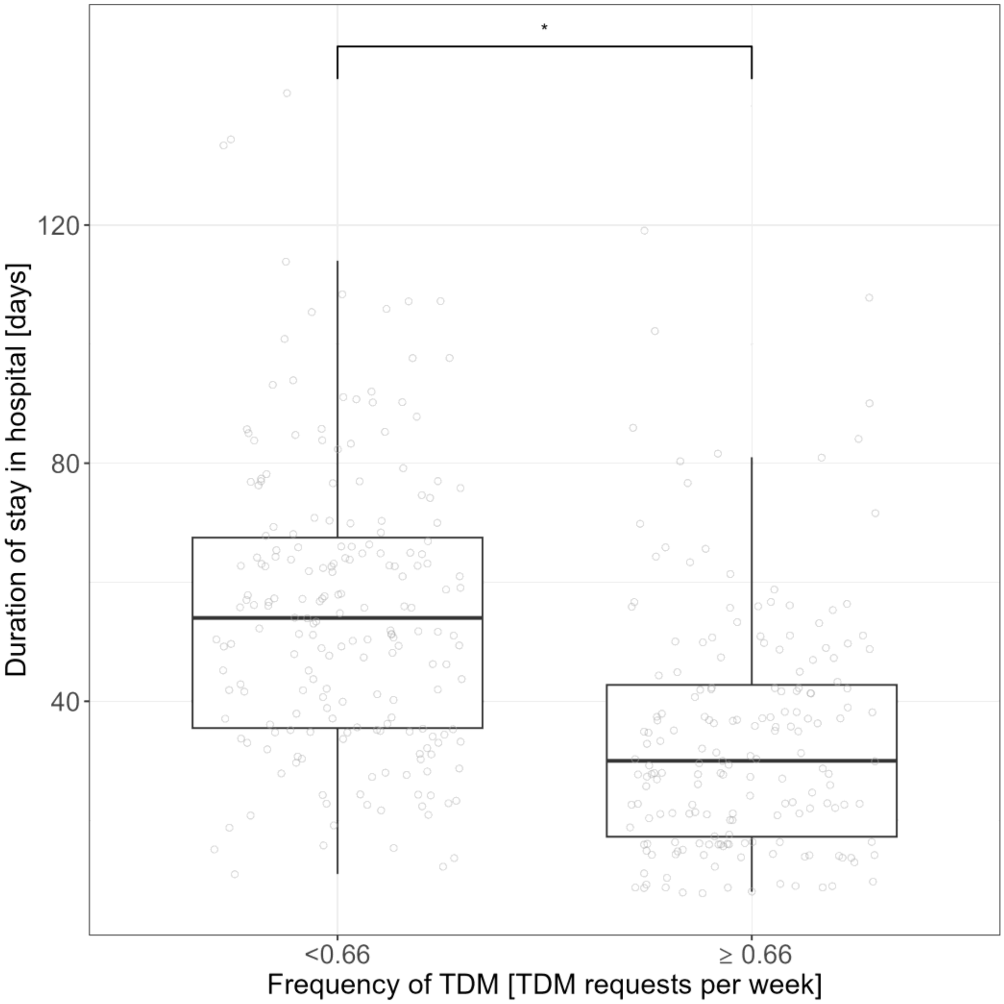

Supplemental Figure 7

TDM frequency for amitriptyline was positively associated with the number of dose adaptions ($p=5.83*10^{-4}$) within 7 days after TDM analysis per hospitalization week. Point transparency reflects local data density, with darker points indicating a higher number of overlapping observations.

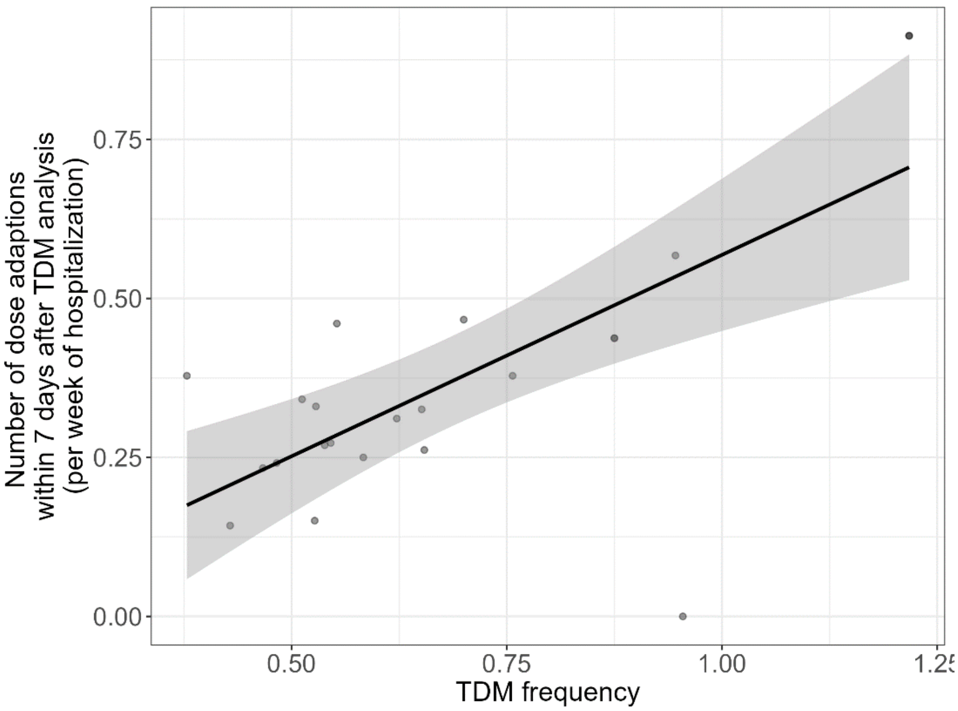

Supplemental Figure 8

TDM frequency for venlafaxine was positively associated with the number of dose adaptions ($p=6.90*10^{-4}$) within 7 days after TDM analysis per hospitalization week. Point transparency reflects local data density, with darker points indicating a higher number of overlapping observations.

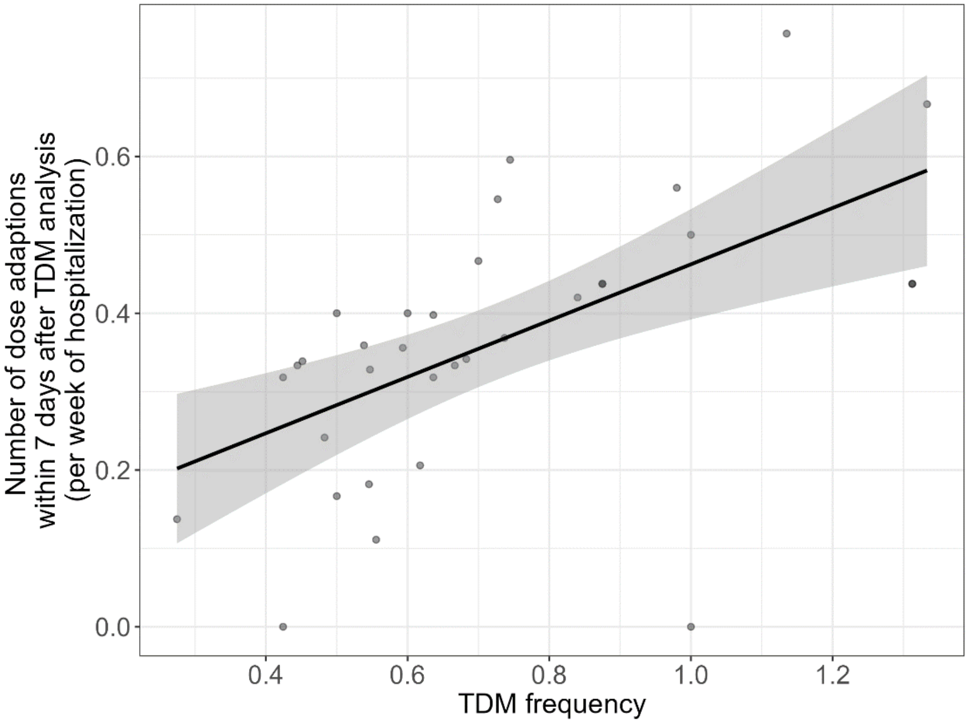

Supplemental Figure 9

TDM frequency for mirtazapine was positively associated with the number of addition/withdrawal of a psychotropic drug (p=0.009) within 7 days after TDM analysis per hospitalization week. Point transparency reflects local data density, with darker points indicating a higher number of overlapping observations.

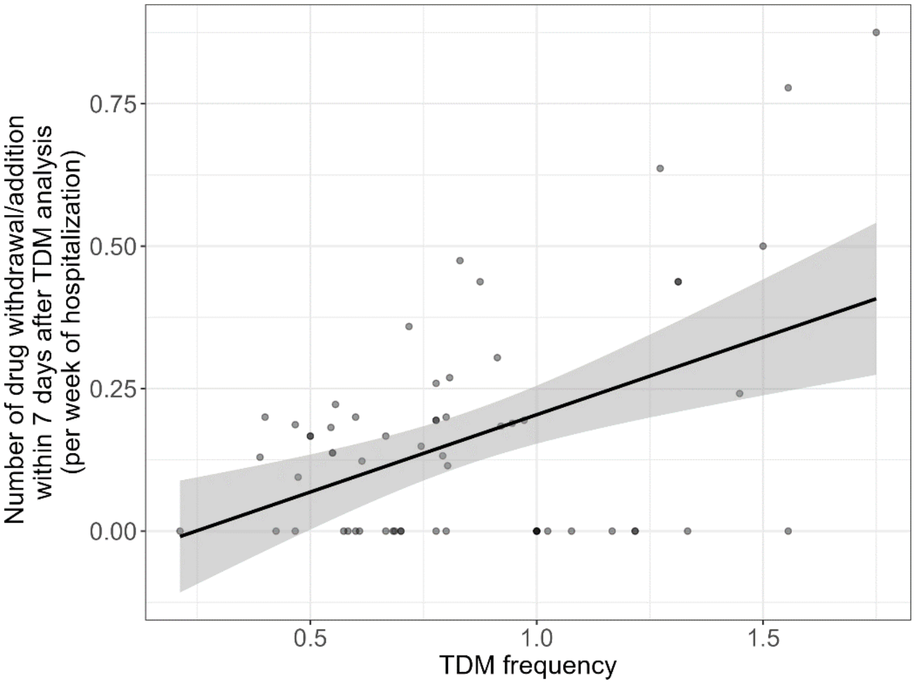

Supplement: Supplementary file 1 — Electronical Supplement [file 41398_2026_4316_MOESM1_ESM.pdf]
